# Factors determining the number of sessions in successful extracorporeal shock wave lithotripsy patients

**DOI:** 10.1515/med-2025-1276

**Published:** 2025-09-11

**Authors:** Müslüm Ergün, Süleyman Sağır

**Affiliations:** Urology Clinic, Medicine Hospital Atlas University, 34200, İstanbul, Turkey; Urology Clinic, Artuklu University, Mardin, 47100, Turkey

**Keywords:** extracorporeal shockwave therapy, session count, urolithiasis

## Abstract

**Background and aim:**

The aim of this study is to investigate whether certain clinical nomograms influencing the success of extracorporeal shock wave lithotripsy (ESWL) also play a role in determining the number of treatment sessions required in patients who achieved stone-free status following ESWL.

**Methods:**

The data of 354 patients with successful ESWL outcomes were analyzed. Patients were evaluated 4–6 weeks post-ESWL using X-ray, ultrasound, or computed tomography. The presence of residual stones larger than 4 mm was considered a treatment failure. Data recorded included age, gender, laterality of stone localization (right/left), stone location (renal pelvis, mid calyx, upper calyx, proximal ureter, mid ureter, distal ureter), stone size (maximum longitudinal dimension), body mass index (BMI), and stone Hounsfield unit (HU) values. Patients were categorized into two groups: single-session and multiple-session treatment.

**Results:**

In the univariate model, factors such as age, BMI, distal ureter, renal pelvis, mid-calyx stone localization, stone size, and stone HU value showed a significant (*p* < 0.05) effect in distinguishing between single-session and multiple-session groups. Stone size and HU values were significantly higher (*p* < 0.05) in the multiple-session group compared to the single-session group. In the multivariate model, age, stone size, and HU value emerged as significant independent factors (*p* < 0.05) in differentiating between single-session and multiple-session treatments.

**Conclusion:**

Several factors influencing the success of ESWL also affect the number of sessions required. BMI, age, stone size, stone HU value, and certain stone locations are key determinants of the number of ESWL sessions.

## 1 Introduction

Despite advancements in laser technologies and endoscopic tools, extracorporeal shock wave lithotripsy (ESWL) remains a significant non-invasive method for the treatment of urinary stone disease. Currently, it is considered the first-line treatment for kidney and proximal ureter stones smaller than 2 cm [1,2]. Modern urologists utilize a wide range of techniques in the management of urolithiasis, including ESWL, ureteroscopy, percutaneous nephrolithotomy (PCNL), retrograde intrarenal surgery (RIRS), as well as open or laparoscopic procedures.

While ESWL holds a prominent place in the treatment of urinary stones, its success rates are generally lower compared to more invasive techniques such as PCNL, RIRS, flexible, and rigid ureterorenoscopy [3,4]. Reported success rates for ESWL in the literature range from 82 to 91% [5,6]. Variations in these rates may stem from differences in inclusion and exclusion criteria or clinical limitations. Numerous factors influencing ESWL success have been identified in the literature, leading to the development of various clinical nomograms.

These nomograms consider parameters such as urinary system anatomy, stone size, location, composition, body mass index (BMI), skin-to-stone distance (SSD), ESWL equipment used, hydronephrosis, and stone Hounsfield unit (HU) values [7,8,9]. By incorporating these factors, clinical nomograms aim to predict stone-free rates (SFR) and assess the likelihood of ESWL success.

The aim of this study is to investigate whether certain clinical nomograms influencing ESWL success also have an impact on the number of treatment sessions required in patients who achieved stone-free status following ESWL.

## 2 Materials and methods

Between January 2022 and March 2024, adult patients presenting to our outpatient clinic with kidney stones were included in the study after retrospective data collection. Patients with stones measuring 5–20 mm who consented to treatment underwent ESWL. Patients excluded from the study included those with unsuccessful ESWL outcomes (residual stones larger than 4 mm), those with double-J stents, stones larger than 20 mm, lower pole kidney stones, or BMI ≥ 35. Data from 354 patients with successful ESWL outcomes were analyzed.

Patients were evaluated 4–6 weeks after ESWL using X-ray, ultrasound (US), or computed tomography (CT). Residual stones larger than 4 mm were defined as treatment failure. In addition, all patients included in the study were given alpha blockers for 1 month. Parameters recorded included age, gender, stone laterality (right/left), stone localization (renal pelvis, mid calyx, upper calyx, proximal ureter, mid ureter, distal ureter), stone size (maximum longitudinal dimension), BMI, and stone HU values. Patients were categorized into two groups: single-session and multiple-session treatments.

All patients underwent pre-procedural non-contrast helical CT. Laboratory investigations included urinalysis and urine culture. Patients with positive urine cultures received antibiotic therapy based on culture antibiogram results before the procedure. Patients were informed about treatment methods. Each session involved administering 2,000–2,500 shock waves at 80 shocks per minute with a power range of 13–17 kV. Fluoroscopy was used throughout the procedure to ensure optimal stone localization and to monitor treatment response. An Elmed device (Elmed Medical Systems Multimedia Classic/Turkey) was used for ESWL.

### 2.1 Data analysis

Descriptive statistics, including mean, standard deviation, median, minimum, maximum, frequency, and percentage, were used to summarize the data. The distribution of variables was assessed using the Kolmogorov–Smirnov and Shapiro–Wilk tests. For non-normally distributed independent quantitative variables, the Mann-Whitney *U* test was applied. For independent qualitative variables, the Chi-square test was used, and Fisher’s test was applied when Chi-square assumptions were not met. The effect size and cutoff values were analyzed using the ROC curve. Univariate and multivariate logistic regression analyses were performed to assess effect sizes. Statistical analyses were conducted using SPSS version 28.0 (SPSS Inc, Chicago, IL, USA).


**Ethics approval and consent to participate:** The study was conducted with the ethical approval from the University Faculty of Medicine Ethics Committee (E-22686390-050.99-42448) received date: 22/03/2023. All methods were performed in accordance with the relevant guidelines and regulations, and informed consent was obtained from all subjects and/or their legal guardians.

## 3 Results

The study included a total of 354 patients, comprising 96 females and 258 males. The mean age of the patients was 43.8 ± 11.9 years, and the mean BMI was 25.0 ± 4.5. Stones were located on the right side in 46.0% (163) of cases and on the left side in 54.0% (191). The mean stone size was 10.0 ± 4.4 mm, and the mean stone HU value was 770.0 ± 153.9 (Table 1).

**Table 1:** Demographic findings of the study

		Min–Max	Median	Mean value ± SD/*n* (%)		
Age		15.0–83.0	42.0	43.8	±	11.9
Sex	Female			96		27.1%
	Male			258		72.9%
BMI		16.8–39.2	24.3	25.0	±	4.5
	Right			163		46.0%
	Left			191		54.0%
Localization	Proximal ureter			109		30.8%
	Distal ureter			93		26.3%
	Renal pelvis			73		20.6%
	Mid ureter			41		11.6%
	Mid calyx			26		7.3%
	Upper calyx			12		3.4%
Stone size (mm)		5.0–20.0	9.0	10.0	±	4.4
Stone HU		330.0–1260.0	790.0	770.0	±	153.9
Number of sessions	I			176		49.7%
	II			88		24.9%
	III			90		25.4%

The proportion of patients who underwent a single ESWL session was 49.7% (176), while 50.3% (178) required multiple sessions (Tables 1 and 2).

**Table 2:** Comparison of patients undergoing single-session and multiple-session ESWL

		Single session (*n*:176)				Multiple sessions (*n*:178)					
		Mean value ± SD/*n* (%)			Median	Mean value ± SD/*n* (%)			Median	*p*	
Age		41.4	±	11.4	40.0	46.1	±	11.9	45.5	***0.000***	^m^
Sex	Female	47		26.7%		49		27.5%		0.862	^*X*²^
	Male	129		73.3%		129		72.5%			
BMI		24.3	±	4.3	24.1	25.7	±	4.5	25.2	***0.001***	^m^
	Right	79		44.9%		84		47.2%		0.664	^*X*²^
	Left	97		55.1%		94		52.8%			
**Localization**											
Proximal ureter		58		33.0%		51		28.7%		0.381	^*X*²^
Distal ureter		61		34.7%		32		18.0%		***0.000***	^*X*²^
Renal pelvis		21		11.9%		52		29.2%		***0.000***	^*X*²^
Mid ureter		26		14.8%		15		8.4%		0.062	^*X*²^
Mid calyx		7		4.0%		19		10.7%		***0.016***	^*X*²^
Upper calyx		3		1.7%		9		5.1%		0.081	^*X*²^
Stone size (mm)		8.2	±	3.1	7.0	11.9	±	4.7	11.0	***0.000***	^m^
Stone HU		690.0	±	136.9	700.0	849.0	±	126.8	851.5	***0.000***	^m^

^m^Mann-Whitney *u* test/^*X*²^Ki-kare test (Fischer test).

In the group requiring multiple ESWL sessions, the mean age of the patients was significantly higher (*p* < 0.05) compared to the single-session group. Gender distribution did not show a statistically significant difference (*p* > 0.05) between the two groups. BMI was significantly higher (*p* < 0.05) in the multiple-session group compared to the single-session group. Laterality of stone localization (right/left) did not differ significantly (*p* > 0.05) between the two groups (Table 2).

Stone localization in the proximal ureter, mid ureter, and upper calyx did not show significant differences (*p* > 0.05) between the single-session and multiple-session groups. However, distal ureter stone localization was significantly lower (*p* < 0.05) in the multiple-session group compared to the single-session group. Conversely, stone localization in the renal pelvis and mid calyx was significantly higher (*p* < 0.05) in the multiple-session group compared to the single-session group (Table 2).

Stone size and HU values were significantly higher (*p* < 0.05) in the multiple-session group compared to the single-session group (Table 2).

In the univariate model, factors such as age, BMI, distal ureter, renal pelvis, mid-calyx stone localization, stone size, and stone HU values were observed to have a significant effect (*p* < 0.05) in distinguishing between single-session and multiple-session groups (Table 3).

**Table 3:** Univariate and multivariate model analysis

	Univariate model			Multivariate model		
	OR	95% GA	*p*	OR	95% GA	*p*
Age	1.036	1.017–1.055	***0.000***	1.026	1.003–1.049	***0.028***
BMI	1.078	1.026–1.132	***0.003***			
**Localization**						
Distal ureter	2.420	1.479–3.961	***0.000***			
Renal pelvis	0.328	0.188–0.574	***0.000***			
Mid calyx	0.347	0.142–0.847	***0.020***			
Stone size (mm)	1.285	1.200–1.376	***0.000***	1.226	1.142–1.316	***0.000***
Stone HU	1.010	1.007–1.012	***0.000***	1.009	1.007–1.012	***0.000***

Logistic Regression (Forward LR).

In the multivariate model, age, stone size, and HU values emerged as significant independent factors (*p* < 0.05) in differentiating between single-session and multiple-session treatments (Table 3).

**Figure 1:**
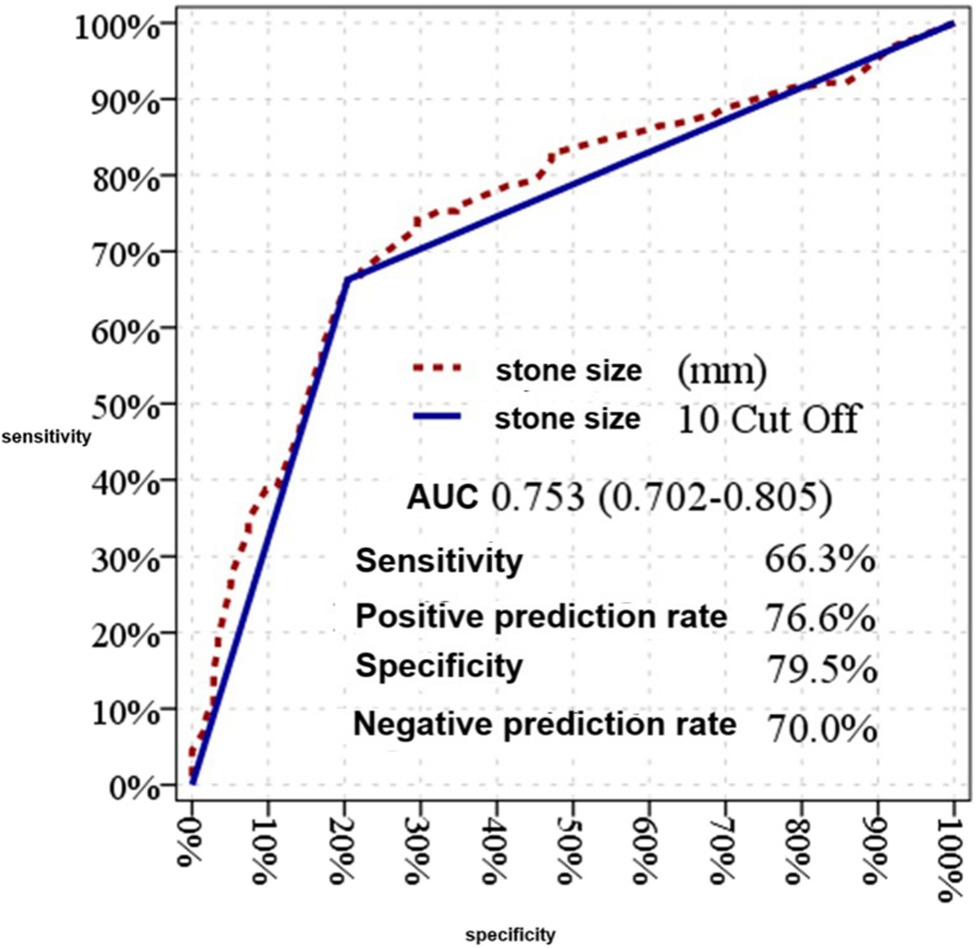
Efficacy graph of stone size between single-session and multi-session patients.

Stone size demonstrated a significant effect in distinguishing between single-session and multiple-session patients, with an area under the curve (AUC) of 0.753 (95% CI: 0.702–0.805) (Table 4). A cutoff value of 10 mm for stone size was observed to have a significant effect, with an AUC of 0.729 (95% CI: 0.676–0.783) (Figure 1). At the 10 mm cutoff value, the sensitivity, specificity, positive predictive value (PPV), and negative predictive value (NPV) for distinguishing between single-session and multiple-session patients were 66.3, 79.5, 76.6, and 70.0%, respectively (Table 4).

**Table 4:** Effectiveness of stone size between single-session and multi-session patients

	AUC	95% CI	*p*
Stone size (mm)	0.753	0.702–0.805	***0.000***
Stone size 10 cut off	0.729	0.676–0.783	***0.000***

		Single session	Multiple sessions				%
Stone size	<10	140	60	Sensitivity			66.3%
	≥10	36	118	PPV			76.6%
				Specificity			79.5%
				NPV			70.0%

ROC Curve.

The HU value of the stone demonstrated a significant effect in distinguishing between single-session and multiple-session patients, with an AUC of 0.801 (95% CI: 0.756–0.846) (Table 5).

**Table 5:** Effectiveness of stone HU value between single-session and multi-session patients

	AUC	95% CI	*P*
Stone HU	0.801	0.756–0.846	***0.000***
Stone HU 755 cutoff	0.737	0.684–0.790	***0.000***

		Single session	Multiple sessions				%
Stone HU	<755	113	30	Sensitivity			83.1%
	≥755	63	148	PPV			70.1%
				Specificity			64.2%
				NPV			79.0%

ROC Curve.

**Figure 2:**
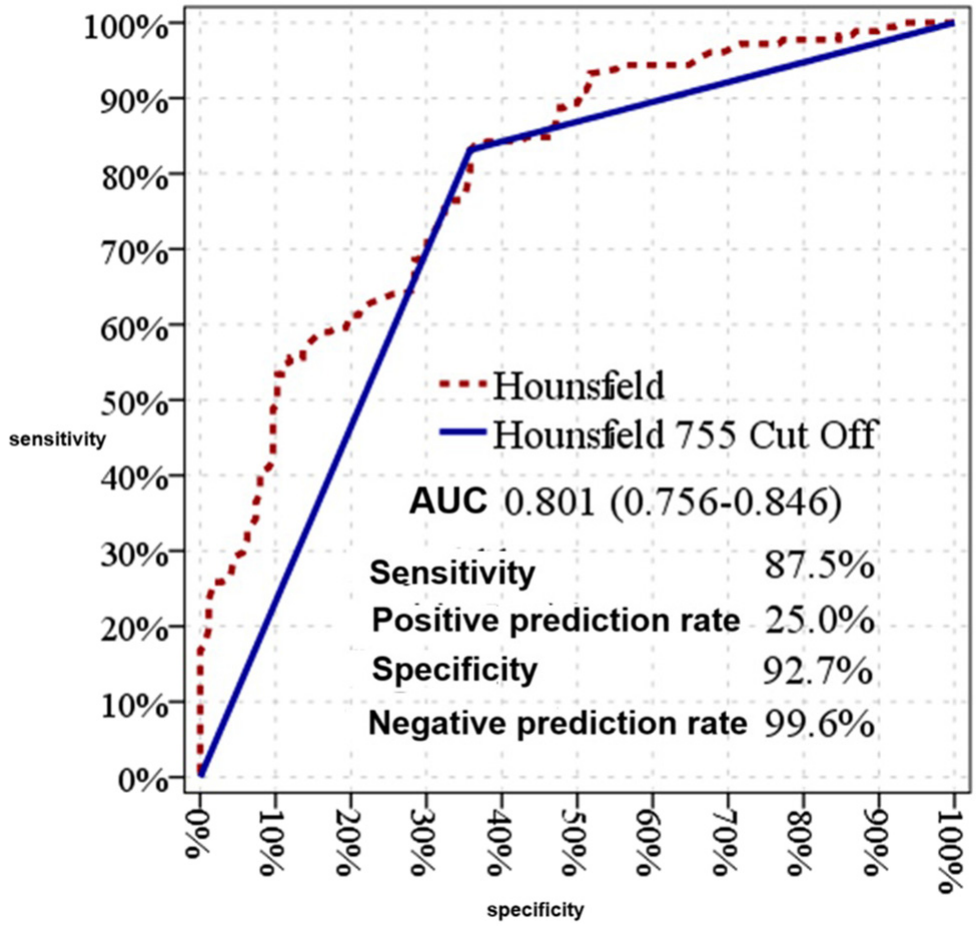
Effectiveness graph of stone HU value in single-session and multi-session patients.

A cutoff value of 755 HU for the stone was also found to have a significant effect, with an AUC of 0.737 (95% CI: 0.684–0.790) (Figure 2).

At the 755 HU cutoff value, the sensitivity, specificity, PPV, and NPV for distinguishing between single-session and multiple-session patients were 83.1, 64.2, 70.1, and 79.0%, respectively (Table 5).

## 4 Discussion

Since the 1980s, ESWL has remained a popular and preferred method for the safe, non-invasive treatment of uncomplicated kidney and ureteral stones (≤20 mm in diameter) [10]. Previous studies have identified predictive factors such as patient age, stone size, stone location, SSD, stone density, severity of obstruction, BMI, urinary system anatomy, and the type of ESWL device, all of which can influence the success of ESWL [11,12]. This study sought to determine whether these predictive factors, in patients who achieved successful outcomes, also impacted the number of sessions required, by categorizing patients into single-session and multiple-session groups.

No significant difference was observed in gender or stone laterality between the groups, suggesting that these factors are not influential in determining the number of ESWL sessions. Similarly, prior research has shown that neither factor significantly affects SFR [13]. Regarding stone location, significant differences were observed between the groups for renal pelvis, mid calyx, and distal ureter stones, while no significant differences were found for stones located in the proximal ureter, mid ureter, or upper calyx. Multiple sessions were more effective for renal pelvis and mid calyx stones, whereas single sessions were more effective for distal ureter stones. Although the literature often associates lower ESWL success rates with distal ureter stones, potentially necessitating more sessions, discrepancies in our results may stem from factors such as stone size, stone HU value, and BMI.

Age and BMI have previously been identified as significant predictors of ESWL success [13,14,15]. In this study, both factors significantly influenced session count in the univariate model, with older age and higher BMI correlating with an increased number of sessions. The mean age of our cohort was 43.8 ± 11.9 years, aligning with the global prevalence of urolithiasis in individuals aged 40–50 years [16]. Although the influence of age diminishes over extended follow-up periods, it remains a strong determinant of SFR after ESWL [17]. Age-related sclerotic changes in renal parenchyma may increase acoustic impedance, leading to lower SFR and higher session counts [14]. Unlike BMI, age remained a significant independent factor in the multivariate model, highlighting its strong impact on session count.

While SSD measurements were not included in this retrospective study, SSD is known to correlate linearly with BMI [18]. Obese patients experience reduced energy transmission to the stone, resulting in decreased SFR [19,20]. In our study, higher BMI was a significant factor in the univariate model but did not demonstrate similar effects in the multivariate model. This aligns with findings from other studies where SSD was a predictor of ESWL failure in univariate analysis but not in multivariate analysis [18].

Both stone size and HU value were significant factors in distinguishing between single-session and multiple-session patients in both univariate and multivariate models. Stone size is a critical variable in determining the appropriateness of ESWL. A prospective study involving 130 patients reported that larger stone sizes required more ESWL sessions [21]. Similarly, other studies have identified a 10 mm cutoff for stone size as an independent predictor of ESWL success [22]. Our study also found a 10 mm cutoff for distinguishing between single-session and multiple-session groups, with an AUC of 0.729 (95% CI: 0.676–0.783). Stones larger than 10 mm necessitate more sessions for successful fragmentation, as corroborated by prior research indicating an average of 1.4 sessions for stones ≤10 mm and 2.1 sessions for stones >10 mm [18].

Stone HU value is another critical factor influencing stone clearance rates. Higher HU values are associated with lower ESWL success and are frequently used to predict treatment outcomes [5,8]. Studies have consistently shown a positive relationship between lower HU values and higher SFR [8,19,23,24]. Evidence from a prospective study demonstrated higher ESWL success rates for stones with HU <970, with a linear relationship observed between stone density and SFR for stones below this threshold [16]. In our study, a 755 HU cutoff was identified as significant in distinguishing between single-session and multiple-session groups, with an AUC of 0.737 (95% CI: 0.684–0.790). This finding underscores the potential of HU values in predicting ESWL success, enabling optimized session planning and cost reduction by minimizing unnecessary sessions.

## 5 Conclusion

Several factors influencing ESWL success also affect the number of sessions required. BMI, age, stone size, stone HU values, and certain stone locations emerged as significant determinants of session count. Among these, stone size and HU value were identified as the most critical independent factors. By considering these variables, the number of ESWL sessions required can be predicted, contributing to the optimization of treatment management.

## 6 Study limitations

This study has several limitations due to its retrospective design. Only sessions of patients who underwent successful ESWL treatment were analyzed, while sessions of those with unsuccessful outcomes were not included, limiting the ability to make comparisons. Additionally, clinical follow-up data did not include measurements for SSD or US shear wave elastography (SWE). Furthermore, data for lower pole kidney stones were insufficient, and therefore, these cases were excluded from the study. As a result, no conclusions regarding session requirements for SSD, SWE, or lower pole kidney stones can be drawn.
